# Post-surgical surveillance in Lynch syndrome--a Cleveland Clinic experience

**DOI:** 10.1186/1897-4287-9-S1-P13

**Published:** 2011-03-10

**Authors:** Susan Fay, Ellen McGannon, James M Church

**Affiliations:** 1 Taussig Cancer Institute, Sanford R. Weiss, M.D. Center of Hereditary Colorectal Neoplasia, Cleveland Clinic, Cleveland, Ohio, USA; 2 Colorectal Surgery Department, Digestive Disease Institute, Sanford R. Weiss, M.D. Center of Hereditary Colorectal Neoplasia, Cleveland Clinic, Cleveland, Ohio, USA

## Background

Post-surgical colonoscopic surveillance is recommended every 1-2 years for patients diagnosed with colon cancer and Lynch syndrome. Patients electing to have a segmental versus colectomy IRA risk developing metachronous colon cancers. The rationale for frequent surveillance is to detect and remove polyps before pathologically advancing to cancer thereby reducing mortality. Does compliance with recommended surveillance eliminate the risk of metachronous cancers?

## Method

A database search for MMR+ families enrolled in the Jagelman Inherited Colorectal Cancer Registry was performed. Patients suitable for this study were status post surgery for colon cancer with the surgery and post operative follow-ups being performed at the Cleveland Clinic.

## Results

**Figure 1:**
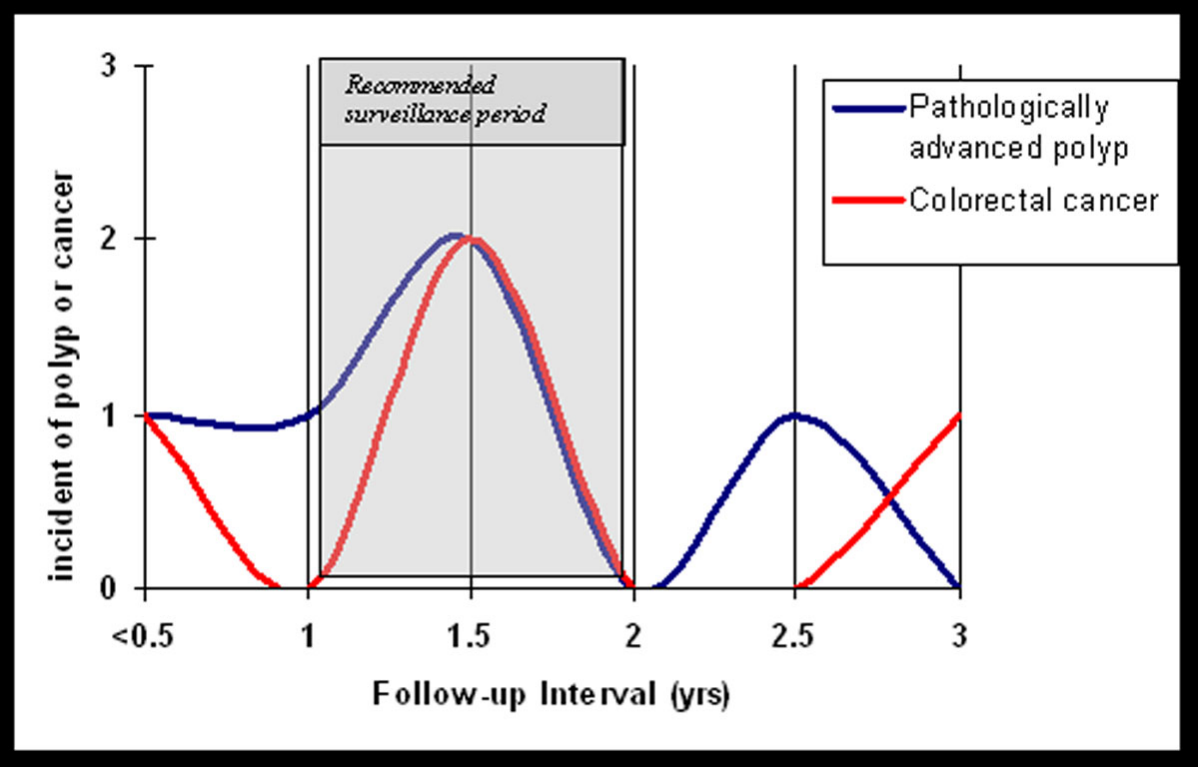


22 patients in 16 families were identified as meeting criteria described above. All but 2 patients are living. One cause of death was advanced colon cancer; the other non-cancer related. Sixteen (64%) segmental resections or hemicolectomies were performed in 14 patients, 3 of the 14 patients had a second surgery for metachronous colon cancer. Colectomy IRAs were performed in 9 patients (36%). Surgeries dated from 1978 to 2010. Collectively, 82 follow-up (FU) colonoscopy, sigmoidoscopy or proctoscopy exams were performed (range 1-9/patient). The interval between surgery and first FU or subsequent FUs totaled 163 years (range 1-22; mean=6.8; median=5). No polyps or cancers were detected in patients having a colectomy IRA. Polyps were detected in 12 out of the 14 (86%) patients who had segmental resections. An analysis of polyp and cancer incidence in patients having segmental resection is shown in Table 1. Figure 1 shows the point within and outside the 1-2 year recommended surveillance period pathologically advanced polyps and cancers were detected.

**Table 1:** Incidence of polyps and cancer following segmental resection

#FU Exams	#FU exams detected polyps	#FU exams detected pathologically advanced polyps*	#FU exams detected cancers	Interval between FU & polyp detected (range= 0.5–4.5 yrs)	Interval between FU & cancer detected (range= 0.5-3 yrs)
52	25 (48%)	7 (13%)	4 (8%)	Mean= 1.5yr Median= 1yr	Mean= 1.4yr Median= 1yr

*pathologically advanced polyp is described as an adenoma being 1cm or larger, having a villous component, or high-grade or severe dysplasia.

Further analysis indicates that if a polyp is detected in a FU exam, it is 28% likely to be pathologically advanced and 16% likely to be cancer.

## Conclusion

1. Compliance with post surgical surveillance guidelines does not eliminate risk of metachronous cancers.

2. Pathologically advanced polyps and cancers can occur within 1 year status post segmental resection.

